# ATG16L1 Depletion-Mediated Activation of the TRAF1 Signaling in Macrophages Aggravates Liver Fibrosis

**DOI:** 10.1155/mi/8831821

**Published:** 2024-11-26

**Authors:** Yufeng Pan, Yi Wei, Xinyu Zhan, Qingfa Bu, Zibo Xu, Xiaozhang Xu, Qi Wang, Yuan Liang, Yue Yu, Haoming Zhou, Ling Lu

**Affiliations:** ^1^School of Medicine, Southeast University, Nanjing, China; ^2^Hepatobiliary Center, Research Unit of Liver Transplantation and Transplant Immunology, Chinese Academy of Medical Sciences, The First Affiliated Hospital of Nanjing Medical University, Nanjing, China; ^3^Department of General Surgery, Nanjing BenQ Medical Center, The Affiliated BenQ Hospital of Nanjing Medical University, Nanjing, China; ^4^School of Biological Science and Medical Engineering, Southeast University, Nanjing, China; ^5^Key Laboratory of Liver Transplantation, NHC Key Laboratory of Hepatobiliary Cancers, Chinese Academy of Medical Sciences, Nanjing, China; ^6^Department of Hepatobiliary and Pancreatic Surgery, Affiliated Hospital of Xuzhou Medical University, 99 Huaihai West Road, Xuzhou, China

**Keywords:** ATG16L1, liver fibrosis, liver inflammation, macrophage, TRAF1

## Abstract

**Background:** Hepatic macrophages play an indispensable role in liver pathophysiology, serving as key orchestrators of both liver injury and repair processes. ATG16L1 (autophagy-related 16 like 1) has emerged as a novel and critical autophagy marker. In macrophages, ATG16L1 assumes a particularly crucial role. The current understanding of how macrophage ATG16L1 regulates liver inflammation in the context of liver fibrosis is unclear.

**Methods:** This study included clinical patient samples of liver fibrosis and established a murine model with myeloid-specific *Atg16l1* knockout, creating a mouse model of liver fibrosis. Employing RNA sequencing, we sought to elucidate the mechanisms of macrophage ATG16L1 in liver fibrosis by identifying critical signaling pathways. To assess the influence of macrophage ATG16L1 on hepatocyte apoptosis and hepatic stellate cell (HSC) activation, we constructed a dedicated culture system. Ultimately, the introduction of mice with myeloid-specific *Atg16l1* knock-in substantiated the protective role of myeloid-specific *Atg16l1* against inflammatory signaling, hepatocyte apoptosis, and activation of HSCs.

**Results:** An upregulation of the ATG16L1 signal was observed in the liver tissues of patients with liver fibrosis and in fibrotic mice, predominantly localized to hepatic macrophages. In *Atg16l1*^Δ*Mφ*^ mice afflicted with liver fibrosis, we detected exacerbated liver damage, evidenced by heightened inflammatory signal expression, increased hepatocyte apoptosis, and enhanced activation of HSCs. The absence of macrophage *Atg16l1* was found to result in elevated TNF receptor-associated factor 1 (TRAF1) signaling, triggering inflammatory activation, intensifying hepatocyte apoptosis, and facilitating HSC activation through the transforming growth factor beta 1 (TGF-*β*1) signaling. The detrimental effects of macrophage *Atg16l1* depletion were demonstrated to be mitigated upon *Atg16l1* reintroduction.

**Conclusions:** This research delved into the mechanisms by which the macrophage ATG16L1 signal influences inflammatory signaling, hepatocyte apoptosis, and activation of HSCs in liver fibrosis. Consequently, it offers theoretical substantiation and an experimental groundwork for the identification of biological targets for therapeutic intervention in liver fibrosis.

## 1. Introduction

Liver fibrosis emerges as a wound-healing response to chronic liver damage triggered by diverse factors such as viral hepatitis, alcohol ingestion, and chemical toxin exposure, marking a pivotal transition point in the progression of chronic liver pathologies toward cirrhosis and hepatocellular carcinoma [1, 2]. Liver fibrosis is a pathological process caused by the continuous accumulation of extracellular matrix (ECM) due to chronic liver injury caused by multiple factors, forming scar tissue. The main manifestation is that hepatic stellate cells (HSCs) are activated and transformed into myofibroblasts (MFs), ECM is excessively deposited, replacing damaged liver tissue [3]. Cell death and inflammation caused by chronic liver injury are two core factors in the development of hepatic fibrosis (HF) [4]. The evaluation from the Global Burden of Disease (GBD) database, spanning from 1990 to 2019 across 204 countries and regions, indicates that cirrhosis continues to be among the leading contributors to disease burden within the 50–74 age demographic, despite a pronounced reduction in its incidence [5]. Globally, liver diseases claim approximately 2 million lives annually, with China's cirrhosis-related mortality representing roughly 11% of the worldwide total [6]. An epidemiological study in 2020 [7] revealed that between 1999 and 2017, the United States witnessed a mortality rate of 12.86 per 100,000 individuals for chronic liver disease, with cirrhosis accounting for 7.96 deaths per 100,000 people. In contrast, China has experienced a significant surge in the number of cirrhosis and chronic liver disease patients, rising from approximately 7 million to almost 12 million [8]. The comprehension of inflammatory signaling pathways in HF is essential for advancing clinical diagnosis, therapeutic strategies, and the development of novel pharmacological agents targeting this condition. HF, a pathological change present in most chronic liver diseases, significantly impacts the physical health and quality of life of patients. Therefore, clinical attention should be paid to the treatment of this condition. Active treatment of liver fibrosis is of great significance in improving patients' quality of life and disease prognosis.

Macrophages play a crucial role in the development and progression of liver fibrosis. Therefore, investigating the specific mechanisms underlying macrophage function in liver fibrosis may provide novel insights and theoretical foundations for clinical interventions and therapeutic strategies. Notably, both Kupffer cells and bone marrow-derived macrophages (BMDMs) exhibit robust expression of transforming growth factor beta (TGF-*β*), underscoring their potential in orchestrating liver fibrosis [9]. TGF-*β* serves as a pivotal regulator during liver fibrosis, activating three mitogen-activated protein kinase (MAPK) signaling pathways, including extracellular signal-regulated kinase, p38, and c-Jun N-terminal kinase, leading to the activation of HSCs [10]. Furthermore, TGF-*β*1 promotes the transition of HSCs to MFs through the activation of the Notch pathway and the upregulation of *α*-smooth muscle actin (*α*-SMA) expression [11].

ATG16L1 (autophagy-related 16 like 1) has emerged as a novel and critical autophagy marker [12]. In macrophages, ATG16L1 assumes a particularly crucial role. Autophagy significantly impacts macrophage function, contributing to the maintenance of intracellular homeostasis. For instance, during inflammatory responses, autophagy provides essential building blocks for the production of inflammatory factors and aids in the clearance of senescent mitochondria to mitigate the harmful effects of excessive ROS. Beyond its role in autophagy regulation, ATG16L1 also modulates LC3-associated phagocytosis [13], thereby implicating it in both autophagy and phagocytosis in macrophages. To validate the activation of autophagy in response to carbon tetrachloride (CCl_4_) intoxication, our analysis of messenger ribonucleic acid (mRNA) expression patterns revealed a significant upregulation of *Atg12*, *Atg5*, and *Atg16l1* transcripts at the fourth and sixth weeks [14]. Currently, the precise mechanisms underlying the regulation of liver inflammation by ATG16L1 during liver fibrosis remain unclear. The present study aims to elucidate the functional role and underlying mechanisms of macrophage ATG16L1 in liver fibrosis, thereby providing novel therapeutic targets and theoretical foundations for clinical interventions.

## 2. Methods

### 2.1. Human Liver Samples

The study cohort comprised liver tissue specimens obtained via percutaneous biopsy from a cohort of 40 patients diagnosed with liver fibrosis at the First Affiliated Hospital of Nanjing Medical University. This tissue was utilized for mRNA analysis (*n* = 40), with a subset (*n* = 6) processed for protein analysis through paraffin embedding. A control group was established using liver specimens from 40 nonalcoholic steatohepatitis (NASH) individuals who had undergone surgery for hepatic hemangioma at the same institution (Table S1).

Exclusion criteria for the control group included a history of diabetes mellitus, alcohol abuse, viral hepatitis, or other hepatic disorders. Each liver specimen underwent a rigorous histopathological evaluation conducted by two board-certified pathologists. The assessment was based on established criteria for hepatic inflammation grading (G) and fibrosis staging (S), as per the Scheuer scoring system. Ethical approval for this investigation was granted by the Institutional Ethics Committee of the First Affiliated Hospital of Nanjing Medical University, with written informed consent secured from all participants prior to liver biopsy or surgical procedures. Table S1 delineates the demographic and clinical profiles of the enrolled liver fibrosis patients. The research was conducted in strict adherence to prevailing governmental regulations and the ethical guidelines as stipulated in the Declaration of Helsinki.

### 2.2. Animals and Treatments

Wild-type (WT), *FloxP-Atg16l1* (*Atg16l1*^*FL/FL*^), *Lyz2-Cre Atg16l1* knockout (*Atg16l1*^Δ*Mφ*^) 6−8-week-old male mice on a C57BL/6 background were used in the experiments. The floxed allele was bred into homozygosity to generate *Atg16l1*^*FL/FL*^ mice. *Atg16l1*^Δ*Mφ*^ mice were generated by crossing *Atg16l1*^*FL/FL*^ mice with *Lyz2-Cre* mice (all on C57BL/6 background). Littermates with floxed alleles but without Cre were used as WT controls (*Atg16l1*^*FL/FL*^). Male 6–8 week-old *Atg16l1*^*FL/FL*^ and *Atg16l1*^Δ*Mφ*^ mice were feeding with normal chow. These mice were randomly assigned to four groups, two of which received intraperitoneal injections of olive oil and carbon tetrachloride (CCl_4_) [14–16] (10% in olive oil, 2 mL/kg, twice a week for 8 weeks) [17] (Figure S1). CCl_4_, recognized as a potent hepatotoxin, induces centrilobular hepatic necrosis and triggers the release of proinflammatory and profibrotic cytokines within the hepatic microenvironment. The metabolic activation of CCl_4_ in the liver further exacerbates these effects, leading to the progression of liver fibrosis and, if exposure is sustained over time, potentially culminating in cirrhosis [18]. For the bile duct ligation (BDL) study, mice were randomly assigned to two distinct cohorts: the sham-operated group and the BDL group (Figure S1). For anesthesia, isoflurane was administered via inhalation, with an induction concentration set at 3% and a maintenance concentration of 1.5%. Within the BDL group, a precise abdominal incision of approximately 2 cm was executed using microdissecting scissors, facilitating exposure of the bile duct. The bile duct was then meticulously doubly ligated with a 4-0 silk suture [19]. Conversely, in the sham-operated animals, the bile duct was exposed without ligation, serving as the control group. After undergoing a 2-week period of normal dietary feeding, the mice in the two surgical groups were euthanized for further analysis. All the mice were housed under specific ventilated, pathogen-free, and thermostatic conditions with a 12-h light–dark cycle at 24°C. Food intake was measured over 1 week in a regular cage using racks, which were weighed every day, and daily food intake was calculated as kcal/day within the time shown. The mice underwent fasting at the end of the experiments. Then, serum and tissues were harvested as described in a previous study [20, 21]. All the animal studies were performed according to the guidelines of the Institutional Animal Use and the Animal Experimentation Ethics Committee of Nanjing Medical University.

### 2.3. Histology, Immunohistochemistry, Immunofluorescence Staining

The liver tissues were fixed with formalin and then embedded in paraffin. The samples were cut into a thickness of 4 μm and stained with hematoxylin and eosin (H&E), Sirius Red, and Masson for histopathological analysis by optical microscope [17]. The expression of a-SMA was assessed through immunohistochemical staining. Hepatocellular tissues were subjected to a series of preparatory processes, including dehydration, clarification, and impregnation with wax, utilizing a biological tissue dehydrator. Following these steps, antigen retrieval and serum sealing were conducted subsequent to the sectioning and baking of the tissue samples. The specimens were then incubated with a primary antibody, followed by a secondary antibody. Subsequently, the slides underwent nuclear staining with 4',6-diamidino-2-phenylindole (DAPI). Detailed antibodies are listed in Table S2.

### 2.4. RNA Isolation and Library Preparation

Total RNA was extracted by employing the TRIzol reagent (Invitrogen, CA, USA) in strict adherence to the manufacturer's protocol. The purity and quantity of the extracted RNA were meticulously assessed using the NanoDrop 2000 spectrophotometer (Thermo Scientific, USA). Additionally, RNA integrity was meticulously evaluated through the utilization of the Agilent 2100 Bioanalyzer (Agilent Technologies, Santa Clara, CA, USA). Subsequently, the libraries were meticulously constructed with the assistance of the VAHTS Universal V6 RNA-seq Library Prep Kit, adhering to the manufacturer's explicit instructions. The transcriptome sequencing and analysis were diligently performed by OE Biotech Co., Ltd. (Shanghai, China).

## 3. Statistical Analyses

All data were analyzed using a two-tailed Student's *t*-test or a one-way analysis of variance (ANOVA), followed by post hoc *t*-tests for multiple comparisons. The data are presented as mean ± standard deviation (SD). Statistical significance was established at a *p*-value less than 0.05 (two-tailed). The statistical analyses were conducted with the aid of GraphPad Prism Version 10.1.2. Statistical significance was determined by a *p* value threshold of less than 0.05. The symbols used to denote statistical significance are asterisks, with the following designations: *⁣*^*∗*^ indicates *p*  < 0.05, *⁣*^*∗∗*^ indicates *p*  < 0.01.

For additional information on materials and methods, please refer to the Supporting Information.

## 4. Results

### 4.1. Macrophage ATG16L1 Signaling Is Closely Related to HF Injury

**Figure 1:**
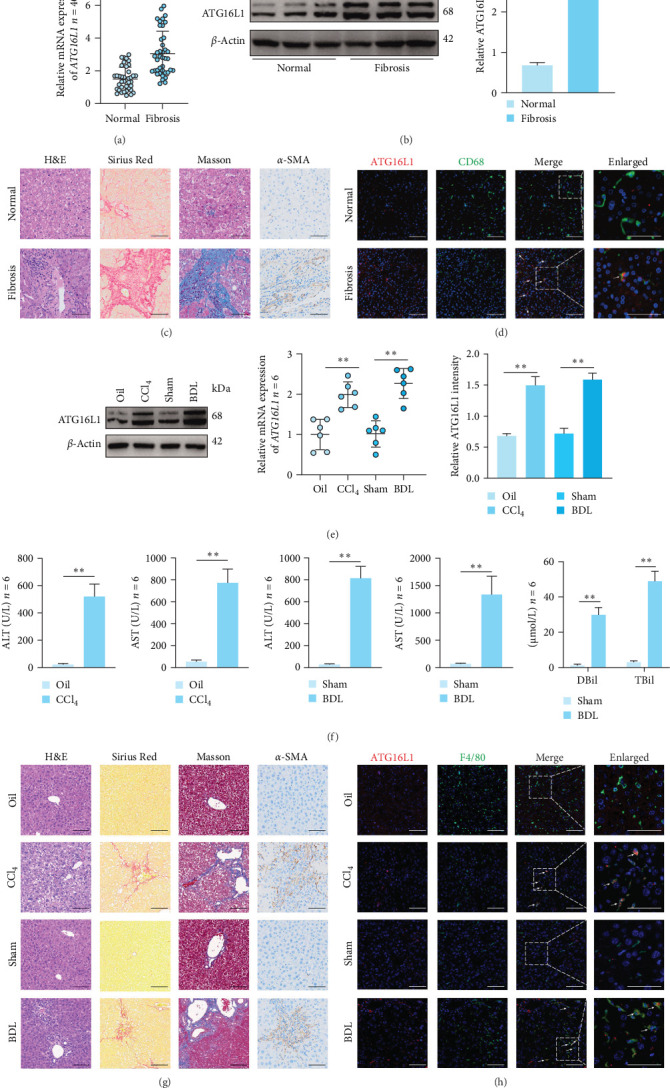
ATG16L1 is upregulated in the liver tissues of patients and mice with HF. (A) The mRNA expression levels of *ATG16L1* in liver tissues from patients with HF (*n* = 40) and normal controls (*n* = 40). (B) The protein levels of ATG16L1 in the liver tissues of normal controls or patients with HF were examined by western blot. (C) Representative H&E staining, Sirius Red staining, Masson staining, and immunohistochemistry images of *α*-SMA (400x) in the liver tissues from patients with HF and normal controls; *n* = 6/group. (D) Dual immunofluorescence staining for CD68 and ATG16L1 (400x) in liver tissues from patients with HF and normal controls; *n* = 6/group. (E) The protein and the mRNA expression levels of ATG16L1 in the liver tissues of mice with HF induced by CCl_4_ or BDL compared to their respective control groups. (F) Serum ALT and AST levels of mice with HF induced by CCl4 or BDL compared to their respective control groups, serum DBil, and TBil levels of BDL and sham surgery group mice. (G) Representative H&E staining, Sirius Red staining, Masson staining, and immunohistochemistry images of *α*-SMA (400x). (H) Dual immunofluorescence staining for F4/80 and ATG16L1 (400x); *n* = 6/group. Data are expressed as mean ± SD. *⁣*^*∗*^*p*  < 0.05, *⁣*^*∗∗*^*p*  < 0.01 (unpaired *t* test or ANOVA). ALT, alanine aminotransferase; ANOVA, analysis of variance; AST, aspartate aminotransferase; ATG16L1, autophagy-related 16 like 1; BDL, bile duct ligation; CCl4, carbon tetrachloride; DBil, direct bilirubin; HF, hepatic fibrosis; mRNA, messenger ribonucleic acid; TBil, total bilirubin.

To elucidate the role of ATG16L1 signaling in HF, we initially assessed the expression levels of ATG16L1 in liver tissues obtained from a cohort of 40 patients diagnosed with HF and an equal number of age- and sex-matched normal controls. Quantitative real-time polymerase chain reaction (PCR) analysis revealed that hepatic *ATG16L1* gene expression was markedly elevated in the HF patient group relative to the control cohort (Figure 1A). The primer sequences utilized in this study are detailed in Table S3. The increased expression of ATG16L1 protein in human HF tissues was further validated through western blot analysis (Figure 1B). Representative images obtained from pathological examination in the liver tissues from patients with HF and normal controls were shown in Figure 1C. Utilizing dual immunofluorescence staining, we observed that the increased expression of ATG16L1 was predominantly localized in macrophages within the livers of patients with HF (Figure 1D). Employing western blot analysis, we observed a significant elevation in ATG16L1 protein levels in liver tissues from mice subjected to HF induction using CCl_4_ or BDL, as compared to their respective sham-operated controls. Consistently, real-time PCR analysis revealed significant activation of the *Atg16l1* signaling pathway in injured liver tissues, as compared to those of control livers (Figure 1E). The serological (Figure 1F) and pathological (Figure 1G) results indicate that we have successfully induced a mouse model of HF by utilizing either CCl_4_ or BDL methods. Through the examination of illustrative H&E, Sirius Red, Masson's trichrome, and *α*-SMA immunohistochemistry staining images, a comprehensive assessment of tissue morphology and protein expression was conducted. In comparison to their respective control groups, histopathological staining of mice in the CCl_4_ and BDL groups demonstrated extensive hepatocellular degeneration, fibrous tissue proliferation in the portal areas, inflammatory cell infiltration, and the effacement of local hepatic lobular architecture. Utilizing dual immunofluorescence staining, we observed that the increased expression of ATG16L1 was predominantly localized in macrophages (Figure 1H). These observations suggest that the activation of ATG16L1 signaling in macrophages is highly correlated with liver fibrosis injury.

### 4.2. Myeloid *Atg16l1* Deficiency Promotes the Development of Experimental HF and Inflammation

**Figure 2:**
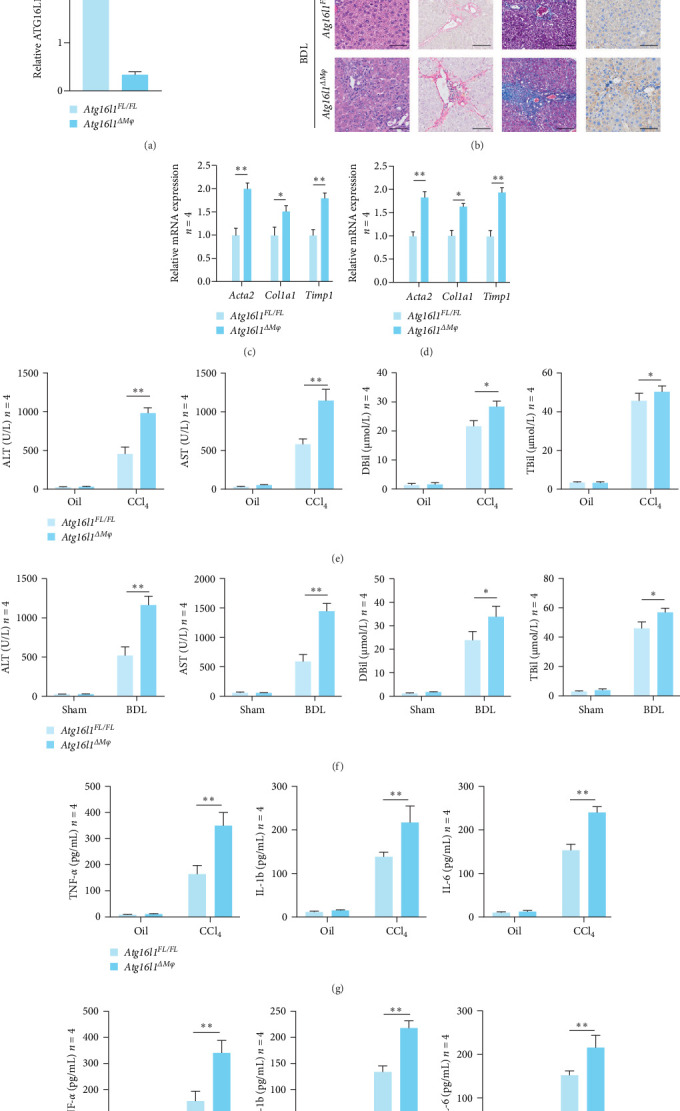
Macrophage *Atg16l1* depletion promotes the development of experimental hepatic fibrosis. (A) The protein levels of ATG16L1 in macrophages isolated from the bone marrow of *Atg16l1*^*FL/FL*^ or *Atg16l1*^Δ*Mφ*^ mice were examined by western blot to verify the knockout efficiency. (B) Representative H&E staining, Sirius Red staining, Masson staining, and immunohistochemistry images of *α*-SMA (400x); *n* = 4/group. (C) The gene expression levels of *Acta2*, *Col1a1*, and *Timp1* in the treated primary in the liver tissues of *Atg16l1*^*FL/FL*^ and *Atg16l1*^Δ*Mφ*^ mice with HF induced by CCl_4_ (C) or BDL (D) were examined by quantitative real-time; *n* = 4/group. (E) Serum ALT, AST, DBil, and TBil levels of *Atg16l1*^*FL/FL*^ and *Atg16l1*^Δ*Mφ*^ mice with HF induced by CCl4 compared to their respective control groups; *n* = 4/group. (F) Serum ALT, AST, DBil, and TBil levels of *Atg16l1*^*FL/FL*^ and *Atg16l1*^Δ*Mφ*^ mice with HF induced by BDL compared to their respective control groups; *n* = 4/group. (G) ELISA analysis of serum TNF-*α*, IL-1b, and IL-6 levels in the *Atg16l1*^*FL/FL*^ and *Atg16l1*^Δ*Mφ*^ mice with HF induced by CCl_4_ (G) or BDL (H); *n* = 4/group. Data are expressed as mean ± SD. *⁣*^*∗*^*p*  < 0.05, *⁣*^*∗∗*^*p*  < 0.01 (unpaired *t* test or ANOVA). ALT, alanine aminotransferase; ANOVA, analysis of variance; AST, aspartate aminotransferase; BDL, bile duct ligation; CCl4, carbon tetrachloride; DBil, direct bilirubin; HF, hepatic fibrosis; IL, interleukin; TBil, total bilirubin.

To assess the effect of macrophage ATG16L1 on the progression of liver fibrosis, we generated myeloid-specific *Atg16l1*-deficient (*Atg16l1*^Δ*Mφ*^) and *Atg16l1*-proficient (*Atg16l1*^*FL/FL*^) mice. To validate the knockout efficiency, western blot analysis was conducted to assess the protein levels of ATG16L1 in macrophages isolated from the bone marrow of *Atg16l1*^*FL/FL*^ and *Atg16l1*^Δ*Mφ*^ mice (Figure 2A). Histochemical staining results have delineated a pronounced exacerbation of hepatic injury in *Atg16l1*^Δ*Mφ*^ mice relative to their *Atg16l1*^*FL/FL*^ counterparts. This is prominently manifested by the pronounced disintegration of hepatic lobular structures and an intensified proliferation of fibrous tissue within the portal triads (Figure 2B). Upon quantitative analysis of the mRNA expression levels of *Acta2*, *Col1a1*, and *Timp1*; we discerned a markedly enhanced activation of HSCs in *Atg16l1*^Δ*Mφ*^ mice subsequent to CCl_4_ (Figure 2C) or BDL-induced injury (Figure 2D), which was substantially higher than that observed in *Atg16l1*^*FL/FL*^ mice. In the CCL_4_-induced murine model of HF, an analysis of serum alanine aminotransferase (ALT) and aspartate aminotransferase (AST) levels (Figure 2E) revealed that *Atg16l1*^Δ*Mφ*^ mice exhibited a more pronounced degree of liver injury in comparison to the *Atg16l1*^*FL/FL*^ mice. Comparable findings were also observed in the BDL-induced liver fibrosis mouse model (Figure 2F). Serum levels of direct bilirubin (DBil) and total bilirubin (TBil) corroborated this finding, further substantiating the observed hepatic injury and dysfunction in the *Atg16l1*^Δ*Mφ*^ mice following CCl_4_ or BDL-induced injury. The enzyme-linked immunosorbent assay (ELISA) results revealed a significant elevation in the levels of serum tumor necrosis factor-alpha (TNF-*α*), interleukin (IL)-1b, and IL-6 upon the disruption of myeloid *Atg16l1*, indicating a heightened inflammatory response (Figure 2G,H). These results further corroborate our finding that inflammatory signaling is being promoted in the absence of Atg16l1 in macrophages.

### 4.3. Macrophage *Atg16l1* Depletion Induces M1 Macrophage Polarization and Activation of Inflammatory Signaling

**Figure 3:**
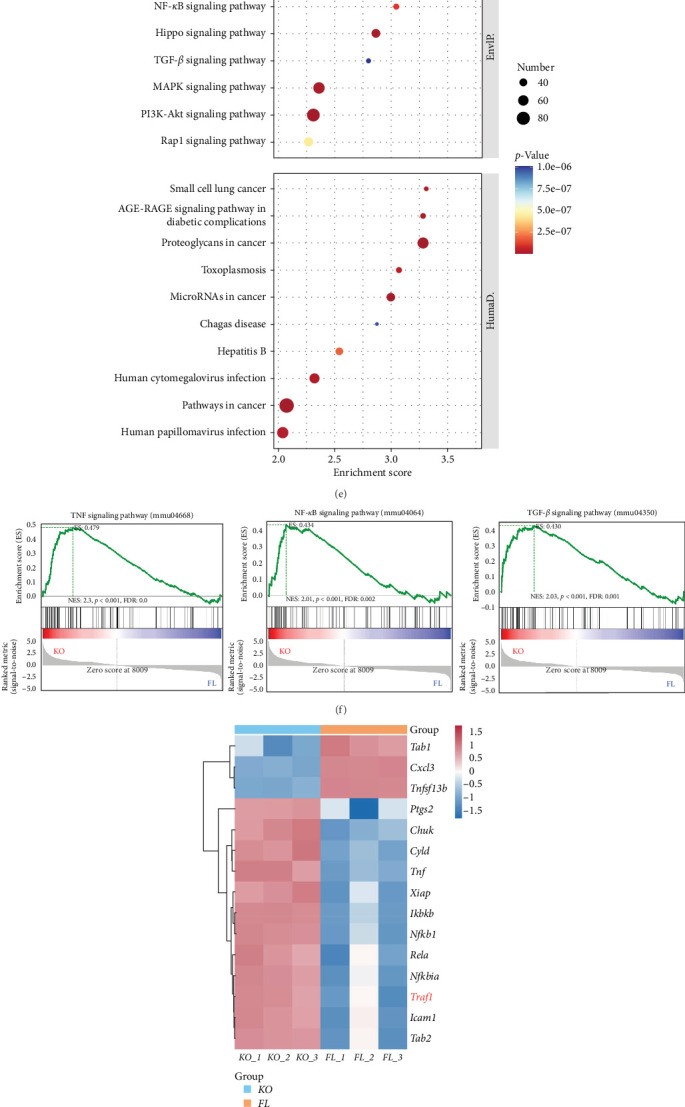
Macrophage *Atg16l1* depletion induces M1 macrophage polarization and activates inflammatory signaling pathways. (A) The protein levels of INOS in macrophages from the bone marrow were treated with or without LPS treatment for 24 h in vitro. (B) The mRNA expression levels of *Nos2* in macrophages from the bone marrow were treated with or without LPS (100 ng/mL) treatment for 24 h in vitro. (C) Flow cytometry analysis of M1 (F4/80^+^INOS^+^) macrophages from the bone marrow were treated with LPS for 24 h in vitro. (D) The mRNA expression levels of pro-inflammatory cytokines *Tnfa*, *Il6*, and *Il1b*, and anti-inflammatory cytokines *Il10*. (E) BMDMs from *Atg16l1*^*FL/FL*^ and *Atg16l1*^Δ*Mφ*^ mice were subjected to RNA sequencing analysis after 24 h of incubation with LPS; *n* = 3/group. The top 20 enriched pathways from the KEGG enrichment analysis are listed. (F) GSEA analysis of TNF signaling pathway, NF-*κ*B signaling pathway, and TGF-*β* signaling pathway. (G) The gene expression levels of *Traf1* in LPS-primed *Atg16l1*^*FL/FL*^ and *Atg16l1*^Δ*Mφ*^ BMDMs were examined by quantitative real-time PCR; *n* = 3/group. (H) Western blot analysis to evaluate the protein expression levels of TRAF1 and NF-*κ*B p65 in hepatic macrophages isolated from *Atg16l1*^*FL/FL*^ and *Atg16l1*^Δ*Mφ*^ with HF. (I) The mRNA expression levels of *Traf1* (I) and RELA (NF-*κ*B p65) (J) in hepatic macrophages isolated from *Atg16l1*^*FL/FL*^ and *Atg16l1*^Δ*Mφ*^ with HF. (K) The protein levels of TRAF1 and NF-*κ*B p65 of BMDMs treated with LPS and TNF-*α* inhibitor for 24 h in vitro. Data are expressed as mean ± SD. *⁣*^*∗*^*p*  < 0.05, *⁣*^*∗∗*^*p*  < 0.01 (unpaired *t* test or ANOVA). ANOVA, analysis of variance; ATG16L1, autophagy-related 16 like 1; BMDM, bone marrow-derived macrophage; HF, hepatic fibrosis; KEGG, Kyoto Encyclopedia of Genes and Genomes; LPS, lipopolysaccharide; mRNA, messenger ribonucleic acid; NF, nuclear factor; RELA, v-Rel Avian Reticuloendotheliosis Viral Oncogene Homolog A; TRAF1, TNF receptor-associated factor 1.

Macrophages are integral components of the innate immune system, and their activation has been demonstrated to be indispensable in various aspects, such as immune defense, inflammatory responses, tissue remodeling, and homeostasis [22]. Upon stimulation by different factors, macrophages differentiate into distinct phenotypes, exhibiting varied characteristics and functions, thereby exerting diverse regulatory roles in the physiological and pathological activities of the body. This phenomenon is also referred to as the polarization effect of macrophages [23]. Macrophages are categorized into pro-inflammatory M1 and anti-inflammatory M2 phenotypes. We aimed to determine which of these macrophage subtypes predominantly mediated the function of macrophage *Atg16l1* in the context of liver fibrosis. We conducted an assessment of the protein levels of inducible nitric oxide synthase (iNOS) in macrophages derived from bone marrow following a 24-h in vitro treatment with or without lipopolysaccharide (LPS) at a concentration of 100 ng/mL (Figure 3A). The mRNA expression levels corroborated this finding (Figure 3B). Flow cytometry analysis targeting M1 macrophages, identified by F4/80 and iNOS positivity, revealed that the deficiency of *Atg16l1* in macrophages leads to an increased polarization toward the M1 phenotype (Figure 3C). The mRNA expression levels substantiated that the absence of macrophage *Atg16l1* resulted in elevated expression of pro-inflammatory factors, further confirming the activation of inflammatory signaling pathways (Figure 3D). BMDMs from *Atg16l1*^*FL/FL*^ and *Atg16l1*^Δ*Mφ*^ mice were subjected to RNA sequencing analysis, which was cultured in vitro and exposed to LPS for a duration of 24 h (*n* = 3/group). Kyoto Encyclopedia of Genes and Genomes (KEGG) enrichment analysis revealed the top 20 pathways that exhibited increased expression in BMDMs from *Atg16l1*^Δ*Mφ*^ mice compared to their counterparts (Figure 3E). Gene set enrichment analysis (GSEA) confirmed that the upregulation of the TNF, nuclear factor (NF)-kappa B (NF-*κ*B) and TGF-*β* signaling pathways are differentially significant (Figure 3F). The results showed that macrophage *Atg16l1* knockout upregulates the expression of TNF receptor-associated factor 1(*Traf1*) and v-rel reticuloendotheliosis viral oncogene homolog A (avian) (v-Rel Avian Reticuloendotheliosis Viral Oncogene Homolog A [RELA], also called p65) (Figure 3G). Western blot analysis was employed to evaluate the protein expression levels of TRAF1 and NF-*κ*B p65 in hepatic macrophages isolated from *Atg16l1*^*FL/FL*^ and *Atg16l1*^Δ*Mφ*^ with HF. The results indicate that in mice with liver fibrosis, *Atg16l1*^Δ*Mφ*^ mice exhibit higher expression levels of TRAF1 and NF-*κ*B p65 in their macrophages compared to the *Atg16l1*^*FL/FL*^ mice (Figure 3H–J). The interplay between TRAF1 and NF-*κ*B is pivotal for the orchestration of cellular signaling events. In the NF-*κ*B signaling axis, TRAF1 is implicated in signal propagation through a spectrum of mechanisms. Specifically, TRAF1 engages with the cytoplasmic region of the B-cell activating factor (BAFF) receptor, functioning as an agonistic modulator within the NF-*κ*B signaling network [24–26]. We examined the expression profiles of TRAF1 and NF-*κ*B p65 in BMDMs isolated from *Atg16l1*^*FL/FL*^ and *Atg16l1*^Δ*Mφ*^ mice after 24 h of in vitro culture, either in the presence or absence of LPS stimulation. Upon the targeted suppression of TRAF1 using a specific TNF-*α* inhibitor, we discerned a significant attenuation in the expression levels of NF-*κ*B p65 (Figure 3K).

### 4.4. Macrophage *Atg16l1* Deficiency Leads to the Activation of Inflammatory Signaling in Liver Fibrosis, Further Exacerbating Hepatocyte Apoptosis

**Figure 4:**
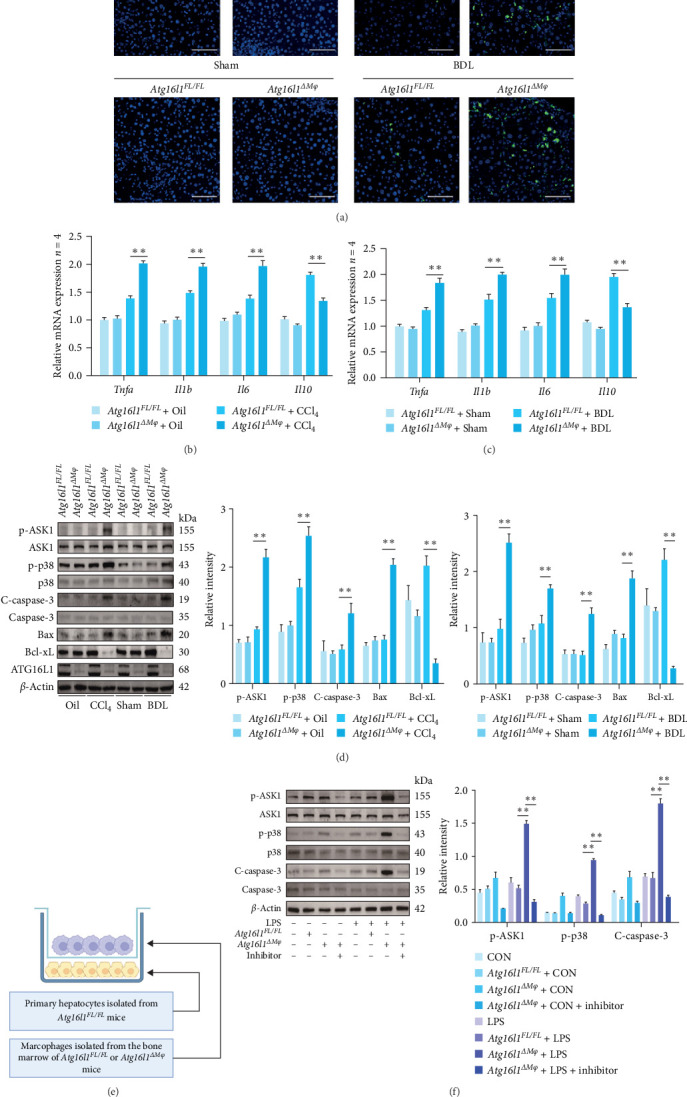
Macrophage Atg16l1 deficiency leads to enhanced inflammatory signaling and aggravated hepatocyte apoptosis. (A) Representative TUNEL staining (400x) images in liver sections from the *Atg16l1*^*FL/FL*^ and *Atg16l1*^Δ*Mφ*^ mice with HF; *n* = 4/group. (B) The hepatic mRNA expression levels of pro-inflammatory cytokines *Tnfa*, *Il6*, and *Il1b*, and anti-inflammatory cytokines *Il10* in *Atg16l1*^*FL/FL*^ and *Atg16l1*^Δ*Mφ*^ mice with HF induced by CCl_4_ (B) or BDL (C). (D) The protein levels of p-ASK1, ASK1, p-p38, p38, C-caspase-3, caspase-3, Bax, and Bcl-xL in the liver tissues of *Atg16l1*^*FL/FL*^ and *Atg16l1*^Δ*Mφ*^ mice with HF were examined by western blot. (E) Schematic of primary hepatocytes from *Atg16l1*^*FL/FL*^ mice cocultured with primary bone marrow-derived macrophages from *Atg16l1*^*FL/FL*^ mice or *Atg16l1*^Δ*Mφ*^ mice with or without LPS and TNF-*α* inhibitor treatment (created with BioRender.com). (F) The protein levels of p-ASK1, ASK1, p-p38, p38, C-caspase-3 and caspase-3 were examined by western blot. Data are expressed as mean ± SD. *⁣*^*∗*^*p*  < 0.05, *⁣*^*∗∗*^*p*  < 0.01 (unpaired *t* test or ANOVA). ANOVA, analysis of variance; ATG16L1, autophagy-related 16 like 1; BDL, bile duct ligation; CCl4, carbon tetrachloride; HF, hepatic fibrosis; LPS, lipopolysaccharide; mRNA, messenger ribonucleic acid.

Viral, bacterial infections, bile acids, alcohol metabolites, and various other hepatotoxic substances have the potential to induce hepatocyte damage and cell death [27]. In damaged hepatocytes, signaling pathways such as NF-*κ*B are activated, inducing the release of proinflammatory cytokines and chemokines such as TNF-*α*, IL-6, and chemokine CC motif ligand 2 (CCL2). This triggers an inflammatory response [2]. Simultaneously, hepatocyte death manifesting as necrosis, necroptosis, and pyroptosis is accompanied by the substantial release of inflammatory mediators. This release not only intensifies the inflammatory response but also stimulates the activation of HSCs and Kupffer cells, ultimately triggering the onset and progression of HF [28]. Utilizing the TUNEL assay, we evaluated the influence of myeloid *Atg16l1* signaling on hepatocellular apoptosis. A significant increase in the incidence of apoptotic TUNEL + cells was noted in the hepatic tissue of *Atg16l1*^Δ*Mφ*^ mice relative to the *Atg16l1*^*FL/FL*^ mice (Figure 4A). Analysis of hepatic mRNA expression levels of pro-inflammatory cytokines in conjunction with the anti-inflammatory cytokine in *Atg16l1*^Δ*Mφ*^ mice with HF induced by CCl_4_ (Figure 4B) or BDL (Figure 4C) revealed a pronounced enhancement in the activation of hepatic inflammatory signaling pathways relative to the *Atg16l1*^*FL/FL*^ mice, indicative of a more robust inflammatory response in the context of macrophage *Atg16l1* deficiency during HF. Furthermore, Western blot analysis demonstrated that the ablation of Atg16l1 in myeloid cells led to an upregulation of phosphorylated ASK1 (p-ASK1), phosphorylated p38 (p-p38), and cleaved caspase-3 (C-caspase-3) in the liver tissues of *Atg16l1*^Δ*Mφ*^ mice with HF induced by CCl_4_ or BDL when compared to the control livers from *Atg16l1*^*FL/FL*^ mice (Figure 4D). This was accompanied by a decrease in the expression of the survival-promoting protein Bcl-xL and a corresponding increase in the expression of the pro-apoptotic protein Bax in the liver tissues of *Atg16l1*^Δ*Mφ*^ mice (Figure 4D). We posit that the absence of *Atg16l1* in macrophages triggers an upregulation of TRAF1, thereby initiating inflammatory signaling cascades that culminate in hepatocyte apoptosis. To substantiate this hypothesis, we performed cocultures of BMDMs from *Atg16l1*^*FL/FL*^ and *Atg16l1*^Δ*Mφ*^ mice, which were subjected to LPS pretreatment or left untreated, alongside primary hepatocytes from *Atg16l1*^*FL/FL*^ mice (Figure 4E). The cocultures were meticulously conducted under conditions that included or omitted LPS and the specific TNF-*α* inhibitor to elucidate the underlying mechanisms. By examining the levels of protein expression, we discovered that the hepatocyte apoptosis triggered by the ablation of myeloid *Atg16l1* in macrophages was mitigated following the suppression of TRAF1. This was manifested primarily by the diminished expression of p-ASK1, p-p38, and C-caspase-3, coupled with an increase in the expression of the survival-promoting protein Bcl-xL, and a concomitant reduction in the expression of the pro-apoptotic protein Bax (Figure 4F).

### 4.5. Macrophage *Atg16l1* Deficiency Potentiates TGF-*β*1-Mediated HSC Activation in Liver Fibrosis

**Figure 5:**
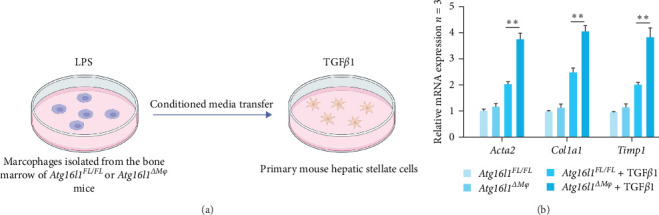
Macrophage *Atg16l1* depletion promotes HSCs activation by stimulating *Tgfb1* expression. (A) Schematic of primary mouse HSCs cocultured with primary bone marrow-derived macrophages from *Atg16l1*^*FL/FL*^ mice or *Atg16l1*^Δ*Mφ*^ mice in the absence or presence of TGF*β*1 (8 ng/mL for 24 h) with LPS treatment (created with BioRender.com). (B) The gene expression levels of *Acta2*, *Col1a1*, and *Timp1* were examined by quantitative real-time; *n* = 3/group. Data are expressed as mean ± SD. *⁣*^*∗*^*p*  < 0.05, *⁣*^*∗∗*^*p*  < 0.01 (unpaired *t* test or ANOVA). ANOVA, analysis of variance; HSCs, hepatic stellate cells; LPS, lipopolysaccharide; TGFβ1, transforming growth factor beta-1.

In our previous studies utilizing RNA sequencing analysis, we detected a pronounced upregulation of the TGF-*β* signaling pathways in macrophages with Atg16l1 deficiency. Given the pivotal role of TGF-*β*1 in the activation of hepatic HSCs during liver fibrosis [11, 29], we developed a targeted culture system for HSCs to explore the potential influence of macrophage Atg16l1 on the activation of HSCs through the TGF-*β*1 signaling axis. BMDMs from *Atg16l1*^*FL/FL*^ and *Atg16l1*^Δ*Mφ*^ mice, following stimulation with LPS, were cultured for 24 h. The resultant conditioned media, containing an array of secreted factors from these macrophages, was subsequently applied to cultures of primary mouse HSCs. “These HSCs were incubated in an environment that was either supplemented with TGF-*β*1 at a concentration of 8 ng/mL or not, for a continuous 24-h period (Figure 5A). Examination of the gene expression profiles of markers indicative of fibrosis revealed that the deletion of *Atg16l1* in macrophages results in a marked promotion of HSCs activation (Figure 5B).

### 4.6. Myeloid-Specific *Atg16l1* Knock-In Alleviates Liver Fibrosis and Suppressing Inflammatory Signaling and HSC Activation

**Figure 6:**
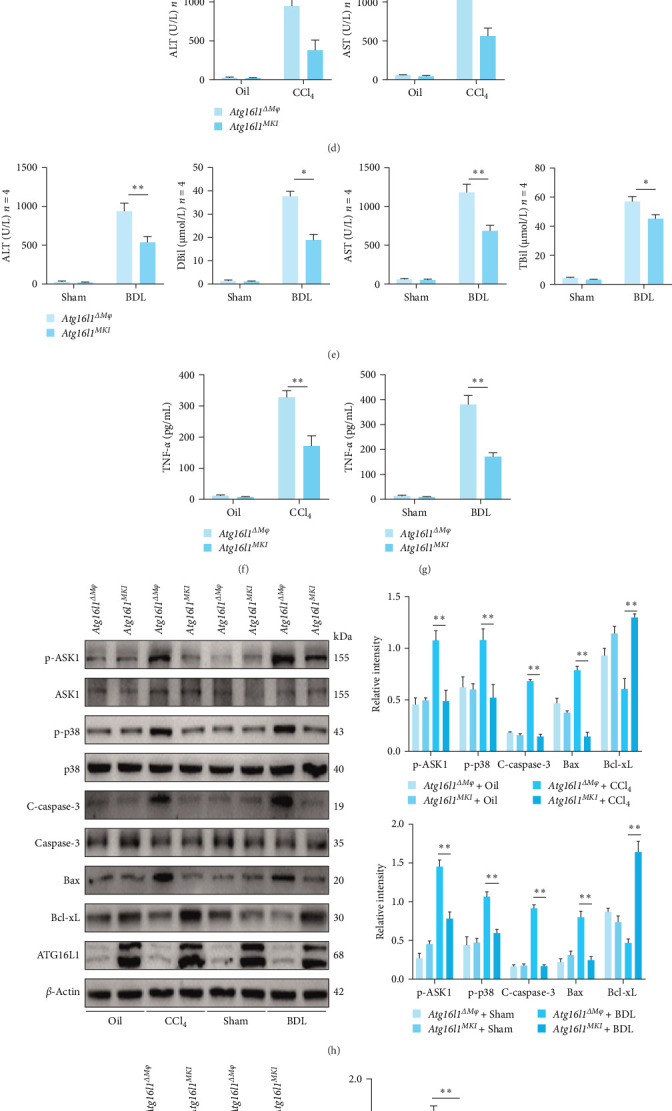
Myeloid-specific knock-in of *Atg16l1* rescues the progression of experimental liver fibrosis caused by its deficiency. (A) Representative H&E staining, Sirius Red staining, Masson staining, and immunohistochemistry images of *α*-SMA (400x); *n* = 4/group. (B) The gene expression levels of *Acta2*, *Col1a1*, and *Timp1* in the treated primary in the liver tissues of *Atg16l1*^Δ*Mφ*^ and *Atg16l1*^*MKI*^ mice with HF induced by CCl_4_ (B) or BDL (C) were examined by quantitative real-time; *n* = 4/group. (D) Serum ALT and AST levels of *Atg16l1*^Δ*Mφ*^ and *Atg16l1*^*MKI*^ mice with HF induced by CCl_4_ compared to their respective control groups; *n* = 4/group. (E) Serum ALT, AST, DBil, and TBil levels of *Atg16l1*^Δ*Mφ*^ and *Atg16l1*^*MKI*^ mice with HF induced by BDL compared to their respective control groups; *n* = 4/group. (C) ELISA analysis of serum TNF-*α* levels in the *Atg16l1*^Δ*Mφ*^ and *Atg16l1*^*MKI*^ mice with HF induced by CCl_4_ (F) or BDL (G); *n* = 4/group. (H) The protein levels of p-ASK1, ASK1, p-p38, p38, C-caspase-3, caspase-3, Bax, and Bcl-xL in the liver tissues of *Atg16l1*^Δ*Mφ*^ and *Atg16l1*^*MKI*^ mice with HF were examined by western blot. (I) The protein levels of TRAF1 and NF-*κ*B p65 of BMDMs treated with or without LPS for 24 h in vitro. Data are expressed as mean ± SD. *⁣*^*∗*^*p*  < 0.05, *⁣*^*∗∗*^*p*  < 0.01 (unpaired *t* test or ANOVA). ALT, alanine aminotransferase; ANOVA, analysis of variance; AST, aspartate aminotransferase; ATG16L1, autophagy-related 16 like 1; BDL, bile duct ligation; BMDM, bone marrow-derived macrophage; CCl4, carbon tetrachloride; DBil, direct bilirubin; HF, hepatic fibrosis; LPS, lipopolysaccharide; NF, nuclear factor; TBil, total bilirubin; TRAF1, TNF receptor-associated factor 1.

Utilizing a myeloid-specific *Atg16l1* knock-in (*Atg16l1*^*MKI*^) mouse model, we noted that histopathological assessments demonstrated attenuated liver damage in *Atg16l1*^*MKI*^ mice relative to *Atg16l1*^Δ*Mφ*^ mice. This was manifested predominantly by a lessened disruption of the hepatic lobular structure and diminished proliferation of fibrous tissue in the portal triads (Figure 6A). In the *Atg16l1*^*MKI*^ mice, our quantitative mRNA expression analysis of fibrotic markers *Acta2*, *Col1a1*, and *Timp1* revealed a pronounced inhibition of HSCs activation in response to CCl4 (Figure 6B) or BDL-induced injury (Figure 6C), in stark contrast to the heightened activation observed in *Atg16l1*^Δ*Mφ*^ mice. This finding suggests a significantly mitigated fibrogenic response in the *Atg16l1*^*MKI*^ mice. In the CCl_4_-induced mouse model of liver fibrosis, assessments of ALT and AST levels indicated a markedly reduced liver injury in *Atg16l1*^*MKI*^ mice (Figure 6D). Consistent observations were made in the BDL-induced model of liver fibrosis. Our serological findings, including DBil and TBil levels, substantiated these results, further validating that the myeloid-specific knock-in of *Atg16l1* mitigated the hepatic injury and dysfunction seen in *Atg16l1*^Δ*Mφ*^ mice (Figure 6E). Additionally, the ELISA data demonstrated a significant decrease in serum TNF-*α* levels post myeloid *Atg16l1* knock-in, as compared to *Atg16l1*^Δ*Mφ*^ mice, signifying a diminished inflammatory response (Figure 6F,G). In western blot analyses, the hepatic tissues of *Atg16l1*^*MKI*^ mice exhibited reduced expression of p-ASK1, p-p38, and C-caspase-3 following CCl_4_-induced or BDL-induced HF, indicating a protective role of myeloid Atg16l1 knock-in against fibrotic signaling pathways, in contrast to the control *Atg16l1*^Δ*Mφ*^ mice (Figure 6H). In our prior studies, we noted an upregulation of TRAF1 and NF-*κ*B p65 in BMDMs from *Atg16l1*^Δ*Mφ*^ mice upon LPS stimulation. This increase was found to be reversed by the myeloid-specific knock-in of *Atg16l1*, as substantiated by western blot analysis (Figure 6I). Collectively, these results indicate that myeloid-specific *Atg16l1* knock-in ameliorates the inflammatory signaling, hepatocyte apoptosis, and activation of HSCs induced by the absence of macrophage *Atg16l1*.

## 5. Discussions

Liver fibrosis is a pathological outcome of the abnormal proliferation of connective tissue within the liver due to various etiologies [30]. It is fundamentally a manifestation of the liver's excessive repair response to chronic injuries [31]. Kupffer cells play a role from the early stages of liver fibrosis; they sense the disruption of liver homeostasis and subsequently express chemokines such as CCL2 and CCL5. These factors facilitate the recruitment of circulating monocytes to the site of liver injury, where they subsequently differentiate into liver-resident macrophages [32]. Liver macrophages demonstrate considerable heterogeneity, representing a diverse population derived from multiple sources. The prevailing literature suggests a predominant polarization of macrophages into two contrasting phenotypes: the pro-inflammatory M1 subtype and its anti-inflammatory counterpart, the M2 subtype [33, 34]. These macrophages contribute to the activation of HSCs through the secretion of fibrogenic cytokines such as TGF-*β* and also promote HSC survival via pro-inflammatory mediators like IL-1*β* and TNF-*α*, thus propelling the fibrotic cascade [29, 35].

First and foremost, our study commenced with clinical samples to analyze the expression levels of ATG16L1 in liver fibrosis. Clinical liver tissue specimens from patients with fibrosis were collected, and the expression levels of ATG16L1 in liver tissues were assessed using methods such as quantitative reverse transcription PCR, western blot, H&E staining, immunohistochemistry, and immunofluorescence.

In the current research, by employing myeloid-specific *Atg16l1*^Δ*Mφ*^ mice, we have discovered a close association between the deficiency of macrophage *Atg16l1* and the exacerbation of liver fibrosis damage. This finding underscores the pivotal role of macrophage *Atg16l1* in maintaining liver health. As a vital component of the immune system, the functional anomalies of macrophages in the liver may directly impact the progression of liver fibrosis. The comparative analysis of hepatic mRNA expression profiles of pro-inflammatory cytokines alongside anti-inflammatory cytokines in *Atg16l1*^Δ*Mφ*^ mice subjected to HF induced by either CCl_4_ or BDL, as opposed to the control *Atg16l1*^*FL/FL*^ mice, has unveiled a significant upregulation in the hepatic inflammatory signaling cascades. This observation underscores the heightened inflammatory response elicited in the setting of macrophage-specific *Atg16l1* deficiency, suggesting a pivotal role for *Atg16l1* in modulating inflammatory processes during the progression of HF. Upon a 24-h exposure to LPS, macrophages with the depletion of *Atg16l1* exhibited a pronounced shift toward an M1-like phenotype. The M1 macrophages, known for their pro-inflammatory and tumoricidal functions, are activated upon LPS stimulation and are capable of producing a spectrum of pro-inflammatory cytokines and chemotactic factors that recruit additional immune cells. These include TNF-*α*, IL-1*β*, IL-12, CCL2, and reactive oxygen species (ROS). The secretion of these mediators is instrumental in exacerbating the inflammatory response during the hepatic injury phase associated with fibrosis progression [23, 36–38]. RNA sequencing analysis has revealed an upregulation of the TNF, NF-*κ*B, and TGF-*β* signaling pathways following the deletion of *Atg16l1* in macrophages. Notably, a significant increase in the expression of *Traf1* was observed in this context. TRAF proteins are pivotal intracellular signaling molecules that exert crucial roles within the immune system. They serve as integral components of signaling cascades initiated by TNFR, Toll-like receptors (TLRs), cytokines, and antigen receptors, thereby orchestrating a myriad of immune responses [39]. TRAF1 plays a significant role in the TNFR signaling pathway by forming a complex with TRAF2. Within this complex, TRAF1 facilitates the activation of the canonical NF-*κ*B pathway. This is achieved through the recruitment of cellular inhibitors of apoptosis proteins (cIAPs) and potentially by enhancing the stability of TRAF2, thereby amplifying the signal transduction process [40]. The NF-*κ*B family, a quintessential group of inducible transcription factors, encompasses NF-*κ*B1 p50, NF-*κ*B2 p52, RELA (commonly denoted as p65), v-Rel Avian Reticuloendotheliosis Viral Oncogene Homolog B (RELB), and c-Rel proto-oncogene, NF-kB subunit, REL proto-oncogene (c-REL) [41]. These proteins are pivotal in the regulation of a plethora of cellular processes, particularly those involved in immune and inflammatory responses, cell survival, and proliferation [42]. Their activation and subsequent translocation to the nucleus upon diverse stimuli underscore their critical role in modulating gene expression and maintaining cellular homeostasis [41, 43]. Through the assessment of mRNA and protein expression levels, we identified a significant upregulation of TRAF1 and NF-*κ*B p65 in the liver tissues of *Atg16l1*^Δ*Mφ*^ mice when juxtaposed with those of the *Atg16l1*^*FL/FL*^ mice. Subsequent in vitro studies utilizing BMDMs isolated from both genotypes demonstrated that exposure to LPS induced a marked increase in the expression levels of TRAF1 and NF-*κ*B p65. To elucidate the role of TRAF1 in the activation of the NF-*κ*B signaling cascade, we employed a TNF-*α* inhibitor to attenuate Traf1 expression. In concordance with our hypothesis, the subsequent downregulation of TRAF1 expression was accompanied by a concomitant decrease in NF-*κ*B p65 levels. These findings suggest a pivotal regulatory role for Traf1 in modulating NF-*κ*B signaling, thereby implicating its potential as a therapeutic target in the context of inflammatory and immune responses.

Considering the well-established role of TGF-*β*1 as a key mediator in the activation of hepatic HSCs during the fibrotic process [11, 35], we sought to delineate the influence of macrophage *Atg16l1* on HSC activation via the TGF-*β*1 signaling axis. These results underscore the intricate relationship between macrophage *Atg16l1* and the regulation of HSC activation, potentially through modulation of the TGF-*β*1 signaling pathway. The observed promotion of HSC activation in the absence of macrophage *Atg16l1* suggests that this gene may act as a negative regulator in the fibrotic response. Furthermore, the use of conditioned media from LPS-stimulated BMDMs provides a more physiologically relevant model to study the crosstalk between macrophages and HSCs. The findings highlight the potential therapeutic implications of targeting macrophage *Atg16l1* or its downstream signaling pathways in the context of liver fibrosis.

In our culminating experiments utilizing the *Atg16l1*^*MKI*^ mouse model, we observed a significant amelioration of liver injury in comparison to the *Atg16l1*^Δ*Mφ*^ mice, accompanied by a reduction in inflammatory levels, diminished hepatocyte apoptosis, and attenuated activation of HSCs. In BMDMs from *Atg16l1*^Δ*Mφ*^ mice stimulated with LPS, there was a pronounced increase in the expression of TRAF1 and NF-*κ*B p65. However, the myeloid-specific knock-in of *Atg16l1* effectively reversed this upregulation. In summary, these findings highlight the therapeutic potential of myeloid-specific *Atg16l1* in alleviating inflammatory signaling, hepatocyte apoptosis, and HSCs activation, thereby presenting a promising avenue for intervention in liver fibrosis.

## 6. Conclusion

We found that the deletion of macrophage Atg16l1 leads to an upregulation of TRAF1 and the activation of the NF-kB pathway, resulting in the inflammatory activation of liver tissues and subsequent hepatocyte apoptosis. A previous study found that [44] lipid stress triggered the interaction between ASK1 and TRAFs, including TRAF1, TRAF2, and TRAF6, which resulted in ASK1 deubiquitination and thereby increased ASK1 protein stability. In our study, coculturing these macrophages with primary hepatocytes demonstrated that the diminished inflammation, consequent to TRAF1 suppression, could rescue hepatocyte apoptosis. In addition to the aforementioned findings, RNA sequencing analysis indicated a pronounced upregulation of Tab2. The Tab2 gene encodes a pivotal signaling adapter protein that exerts its function in a spectrum of physiological processes, encompassing immune responses, regulation of apoptosis, modulation of cell cycle progression, and orchestration of cellular responses to exogenous stimuli, including LPSs [45]. Through interactions with molecules such as TAK1 and TRAF6, TAB2 is implicated in the activation of the NF-*κ*B and MAPK8/JNK signaling pathways, which are integral to the transduction of signals that regulate inflammation and cellular stress responses. Consequently, we posit that macrophage TAB2 may also be a pivotal gene influencing the progression of liver fibrosis. Besides Tab2, we also observed changes in the expression levels of several other genes, such as X-linked inhibitor of apoptosis protein (XIAP). In the context of liver fibrosis, XIAP's role is complex. Furthermore, XIAP has been implicated in the regulation of inflammation through its interaction with NF-*κ*B and other signaling molecules, suggesting a possible role in the inflammatory response observed in liver fibrosis. The interaction of XIAP with these signaling pathways could influence the activation state of HSCs and the subsequent fibrotic response [46]. Emerging evidence suggests that XIAP may also be involved in the modulation of macrophage function [47], a key cellular player in the fibrotic response. Macrophages are instrumental in the removal of cellular debris during tissue repair and the release of inflammatory mediators that can influence fibrosis. The potential involvement of XIAP in macrophage polarization and function could have significant implications for the progression of liver fibrosis.

In conclusion, our study identifies macrophage Atg16l1 as a key regulator in liver fibrosis, with its deletion leading to enhanced inflammation and hepatocyte apoptosis. The crosstalk between macrophages and HSCs through the TGF-*β*1 signaling axis is a critical pathway in the fibrotic response. These findings not only advance our understanding of the cellular and molecular mechanisms underlying liver fibrosis but also offer promising targets for therapeutic intervention.

Future research should explore additional pathways and mechanisms by which macrophage function is modulated in liver fibrosis, including the role of metabolic reprograming, phagocytosis, and the interaction between macrophages and other hepatic cells, such as hepatocytes and sinusoidal endothelial cells. Understanding these complex interactions will be crucial for developing novel therapeutic strategies aimed at resolving inflammation and fibrosis in the liver.

## Data Availability

The data associated with this paper are available upon reasonable request to the corresponding author.

## Supporting Information

Supporting Information Figure S1: schematic illustration of the methodological framework for inducing liver fibrosis in a murine model. Table S1: demographics of all patients' population. Table S2: antibodies (H denotes human and M denotes mice) antibodies used in immunohistochemistry and immunofluorescence staining. Table S3: primers (H denotes human and M denotes mice) primer sequences.
