# Supplementary material for: ATG16L1 Depletion-Mediated Activation of the TRAF1 Signaling in Macrophages Aggravates Liver Fibrosis
Source: Mediators Inflamm. 2024 Nov 26;2024:8831821. doi: 10.1155/mi/8831821 (PMC11614508; doi:10.1155/mi/8831821)
Supplement: Supporting Information — Figure S1: schematic illustration of the methodological framework for inducing liver fibrosis in a murine model. Table S1: demographics of all patients' population. Table S2: antibodies (H denotes human and M denotes mice) antibodies used in immunohistochemistry and immunofluorescence staining. Table S3: primers (H denotes human and M denotes mice) primer sequences. [file 8831821.f1.pdf]

Table of contents

Supplementary materials and methods 2

Fig. S1 4

Table S1 5

Table S2 6

Table S3 7

Supplementary references. 8

Supplementary materials and methods

Cell isolation, culture and treatment

In accordance with our previously published methodology, hepatocytes and liver macrophages were isolated [1]. Bone marrow-derived cells were harvested from the femurs and tibias of male *Atg16l1*$^{FL/FL}$ and *Atg16l1*$^{\Delta M\varphi}$ mice. The marrow was flushed from the osseous cavities using a 1 mL syringe filled with Dulbecco's Modified Eagle Medium (DMEM). The isolated bone marrow cells were subsequently cultured at a density of 3×10^6 cells/mL in DMEM supplemented with 10% fetal bovine serum (FBS) and 20% L929 cell line-derived conditioned medium. This cocktail facilitated the differentiation of the progenitor cells into bone marrow-derived macrophages (BMDMs). By the seventh day of in vitro culture, the adherent cells had fully differentiated into mature macrophages [2 3]. For co-culture studies, primary hepatocytes from male *Atg16l1*$^{FL/FL}$ mice and LPS-primed (100 ng/ml of LPS for 6 h) macrophages from the bone marrow of male *Atg16l1*$^{FL/F}$ or *Atg16l1*$^{\Delta M\varphi}$ mice were seeded on the co-culture chamber (Corning Inc., NY, USA). The co-cultured system was supplied with LPS medium. Co-culture was maintained for a duration of 24 hours. Primary hepatic stellate cells (HSCs) were isolated from the liver of mice [4].

Western blot analysis

Proteins were extracted from liver tissues and cells with ice-cold lysis buffer (50 mM Tris, 150 mM NaCl, 1% sodium deoxycholate, 0.1% sodium dodecyl sulfate, 1% Triton-100). The following primary antibodies were added and incubated with the membranes: ATG16L1 rabbit mAbs (AiFang, Hunan, China); phosphorylated ASK1 (p-ASK1),ASK1, phosphorylated p38 (p-p38),p38 rabbit mAbs(Proteintech, Wuhan, China);Cleaved caspase-3 (C-caspase-3), Caspase-3 xzzxrabbit mAbs(Affinity, Jiangsu, China);β-actin rabbit mAbs (Cell Signaling Technology, MA, USA); Bcl-XL,Bax,NF-kB p65,TRAF1 rabbit mAbs (Abcam, Cambridge, UK). The reactions were detected with HRP-conjugated goat anti-rabbit IgG (Cell Signaling Technology, MA, USA) or goat anti-mouse IgG (Cell Signaling Technology, MA, USA) secondary antibodies.

TUNEL Assay

Apoptotic cells were identified with a One-step TUNEL Apoptosis Detection Ki (KCD-T11007, Crondabio, Shanghai, China). Cells with nuclear-positive staining by fluorescent antibodies for DNA fragmentation were visualized directly by fluorescence microscopy.

Real-time qPCR

Quantitative real-time polymerase chain reaction (qPCR) was employed to assess the expression levels of target genes. Total RNA was extracted from liver tissues and bone marrow-derived macrophages (BMDMs) using TRIzol reagent (Invitrogen, Carlsbad, CA, USA), followed by reverse transcription utilizing a cDNA Synthesis Kit (Roche, Indianapolis, IN, USA). Amplification of the target genes was conducted on a LightCycler 480 system using SYBR Green I Master Mix (Roche, Indianapolis, IN, USA). Each qPCR assay was executed in triplicate to ensure experimental rigor and reliability. Data were normalized to the housekeeping gene β-actin to account for variations in sample loading and integrity. The primer sequences utilized in this study are detailed in Table S3.

Enzyme-linked immunosorbent assay (ELISA)

The levels of TNF-α (Abcam, Cambridge, UK) in serum or cell culture supernatants were quantified with ELISA kits according to the manufacturer's protocols.

Flow cytometry

The specific antibodies or isotype controls used for flow cytometry were as follows: F4/80(BUV737), CD11b (PE) (BD Bioscience, New Jersey, U.S.A.). The cells were examined on a BD FACSCelesta flow cytometer and analyzed using FlowJo 10.1 software.

Figure S1

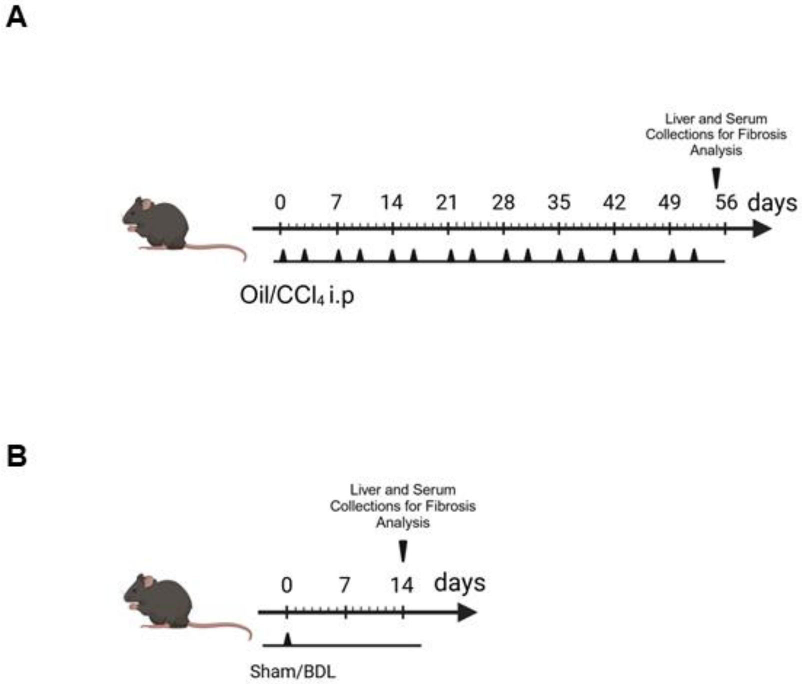

Figure S1. Schematic Illustration of the Methodological Framework for Inducing Liver Fibrosis in a Murine Model. (A) Mice were administered Oil/CCl_4 via intraperitoneal injection twice weekly for a period of eight weeks. (B) Mice were subjected to either BDL or a sham surgical procedure. CCl_4, carbon tetrachloride; BDL, bile duct ligation.

Table S1. Demographics of all patients' population

**Clinical manifestation**	Patients with hepatic hemangioma	Patients with liver fibrosis
Age, years (mean ± SD)	47.39 ± 7.91	52.83 ± 8.47
Gender, n (M/F)	16/24	28/12
BMI, kg/m^2	22.45 ± 5.23	27.97± 4.28
**Laboratory tests** (mean ± SD)		
ALT, U/L	21.14 ± 9.43	69.38 ± 15.71
AST, U/L	24.57 ± 7.52	77.51 ± 12.64
ALB, g/L	42.95 ± 5.14	36.14 ± 6.37
TB, umol/L	7.16 ± 3.89	22.31 ± 6.31
**Histologic characteristics**		
Hepatic inflammation grading (G) 1/2/3/4		11/16/10/3
Fibrosis staging (S)1/2/3/4		8/17/11/4

BMI, Body Mass Index; ALT, Alanine aminotransferase; AST, Aspartate aminotransferase; ALB, Albumin; TB, total bilirubin.

Table S2. Antibodies (H, denotes human and M, denotes mice)

Antibodies used in Immunohistochemistry and Immunofluorescence Staining

Reagent Name	Product Number	Company
H -α-SMA	CAT. #19245S	Cell Signaling Technology, MA, USA
H-Atg16l1	CAT. #8089T	Cell Signaling Technology, MA, USA
H-CD68	ab955	Abcam, Cambridge, UK
M-α-SMA	CAT. #19245S	Cell Signaling Technology, MA, USA
M-Atg16l1	CAT. #8089T	Cell Signaling Technology, MA, USA
M-F4/80	ab300421	Abcam, Cambridge, UK

Table S3. Primers (H, denotes human and M, denotes mice)

Primer sequences

**Gene Symbol**	**Forward Primer**	**Reverse Primer**
H-ATG16L1	TCTGGGACATTCGATCAGAGAG	CCTTTCTGGGTTTAAGTCCAGG
H-β-ACTIN	CATGTACGTTGCTATCCAGGC	CTCCTTAATGTCACGCACGAT
M-Atg16l1	CAGAGCAGCTACTAAGCGACT	AAAAGGGGAGATTCGGACAGA
M-Tnfa	CCTGTAGCCCACGTCGTAG	GGGAGTAGACAAGGTACAACCC
M-Il6	CTGCAAGAGACTTCCATCCAG	AGTGGTATAGACAGGTCTGTTGG
M-Il1b	GAAATGCCACCTTTTGACAGTG	TGGATGCTCTCATCAGGACAG
M-Il10	AAGCTCCAAGACCAAGGTGTC	ACGAGGTTTTCCAAGGAGTTGT
M-Acta2	GTCCCAGACATCAGGGAGTAA	TCGGATACTTCAGCGTCAGGA
M-Col1a1	GCTCCTCTTAGGGGCCACT	CCACGTCTCACCATTGGGG
M-Timp1	CGAGACCACCTTATACCAGCG	ATGACTGGGGTGTAGGCGTA
M-Traf1	TTCAAGGGGAGCCCACAATC	CTCCTTTAAGACCGCCAGCA
M-Rela	GATCGCCACCGGATTGAAGA	TCGGGTAGGCACAGCAATAC
M-β-Actin	GTGACGTTGACATCCGTAAAGA	GCCGGACTCATCGTACTCC

Supplementary references

1. Zhou H, Wang H, Ni M, et al. Glycogen synthase kinase 3beta promotes liver innate immune activation by restraining AMP-activated protein kinase activation. *J Hepatol* 2018; **69**(1): 99-109.

2. Luo X, Li H, Ma L, et al. Expression of STING Is Increased in Liver Tissues From Patients With NAFLD and Promotes Macrophage-Mediated Hepatic Inflammation and Fibrosis in Mice. *Gastroenterology* 2018; **155**(6): 1971-84 e4.

3. Rao J, Cheng F, Zhou H, et al. Nogo-B is a key mediator of hepatic ischemia and reperfusion injury. *Redox Biol* 2020; **37**: 101745.

4. Koo JH, Lee HJ, Kim W, Kim SG. Endoplasmic Reticulum Stress in Hepatic Stellate Cells Promotes Liver Fibrosis via PERK-Mediated Degradation of HNRNPA1 and Up-regulation of SMAD2. *Gastroenterology* 2016; **150**(1): 181-93 e8.
